# Using the Photo–Piezoelectric Effect of AuPt@BaTiO_3_ Oxidase Mimetics for Colorimetric Detection of GSH in Serum

**DOI:** 10.3390/s24072242

**Published:** 2024-03-31

**Authors:** Yiquan Liao, Yichang He, Bin Zhang, Ye Ma, Ruiqi Xu, Minggang Zhao, Hongzhi Cui

**Affiliations:** School of Materials Science and Engineering, Ocean University of China, 266100 Qingdao, China

**Keywords:** AuPt@BaTiO_3_, oxidase–like, colorimetric, photo–piezoelectric, glutathione

## Abstract

Nanozymes possess major advantages in catalysis and biosensing compared with natural nanozymes. In this study, the AuPt@BaTiO_3_ bimetallic alloy Schottky junction is prepared to act as oxidase mimetics, and its photo−piezoelectric effect is investigated. The synergy between the photo−piezoelectric effect and the local surface plasmon resonance enhances the directional migration and separation of photogenerated electrons, as well as hot electrons induced by the AuPt bimetallic alloy. This synergy significantly improves the oxidase−like activity. A GSH colorimetric detection platform is developed based on this fading principle. Leveraging the photo−piezoelectric effect allows for highly sensitive detection with a low detection limit (0.225 μM) and reduces the detection time from 10 min to 3 min. The high recovery rate (ranging from 99.91% to 101.8%) in actual serum detection suggests promising potential for practical applications. The development of bimetallic alloy heterojunctions presents new opportunities for creating efficient nanozymes.

## 1. Introduction

The molecular composition of serum can provide insights into the overall health of an individual. Notably, glutathione (GSH), a potent antioxidant in the human body, plays a crucial role in trapping and neutralizing highly active free radicals [1]. Abnormal levels of GSH have been linked to various diseases, including cancer [2] and Parkinson’s disease [3], making it a valuable biomarker. Current GSH detection methods encompass electrochemical techniques [4], fluorescence spectroscopy [5], and surface−enhanced Raman spectroscopy [6]. Hence, for comprehensive health monitoring, it is imperative to determine GSH concentrations accurately and swiftly.

While natural nanozymes boast remarkable catalytic activity, their applications are often hampered by strict storage prerequisites, prohibitive costs, and limited stability. Consequently, nanozymes have garnered increasing attention. Various enzymatic nanomaterials, such as Fe_3_O_4_ and CuO (serving as peroxidase nanozymes), and MnO_2_ and Ag_2_O (acting as oxidase nanozymes) [7], have been developed. Owing to their cost−effectiveness and stability, they have found applications in fields like food safety and environmental monitoring. Transitioning from natural nanozymes to potent nano−nanozymes can substantially enhance biodetection’s efficacy, a burgeoning trend in bioanalysis. However, many nanozymes are vulnerable to external perturbations, often exhibiting suboptimal catalytic activity. This underscores the need for developing nanozymes that marry high catalytic activity with selectivity.

BaTiO_3_ stands out as a premier piezoelectric material, boasting exceptional chemical and structural robustness [8]. The piezoelectric effects, triggered by mechanical energy or ultrasonic waves, can influence carrier migration and band bending [9]. Concurrently, the local surface plasmon resonance (LSPR) effect inherent to noble metal nanoparticles has been the subject of extensive research [10,11,12]. Given gold’s (Au) stability, its alloys, particularly with platinum (Pt), exhibit enhanced stability and co−catalytic prowess [13]. It is been discerned that the integration of the piezoelectric and LSPR effects can facilitate more efficient photogenerated carrier separation [14]. The juxtaposition of semiconductor materials and noble metals, resulting in a Schottky barrier, is adept at curbing electron−hole pair recombination [15]. This sets the stage for the intriguing proposition of developing bimetallic alloy nanozymes, particularly exploring their photo−piezoelectric impact on catalytic enhancement.

In our current study, we synthesized an AuPt@BaTiO_3_ bimetallic alloy Schottky junction, leveraging it as oxidase mimetics and delving into its photo−piezoelectric characteristics. The interplay between the photo−piezoelectric phenomenon and the LSPR of the bimetallic alloy bolstered the oxidase−like activity significantly. Using the AuPt@BaTiO_3_ sea−urchin microspheres (SUMs), we realized swift and precise GSH detection. Remarkably, the photo−piezoelectric effect slashed the detection duration from 10 min to a mere 3 min. Crafting such bimetallic alloy heterojunctions, with an emphasis on their photo−piezoelectric attributes, offers promising avenues for nano−enzyme development.

## 2. Materials and Methods

### 2.1. Reagents and Materials

All reagents were not purified further before use. Methylbenzene, hydrochloric acid (HCl), lysine (Lys), arginine (Arg), titanium tetrachloride (TiCl_4_), tetrabutyltitanate, gold (III) chloride (HAuCl_4_·3H_2_O), leucine (Leu), cysteine (Cys), proline (Pro), glycine (Gly), barium hydroxide octahydrate (Ba(OH)_2_·8H_2_O), formic acid, ethylene diamine tetraacetic disodium (EDTA−2Na), chloroplatinic acid (H_2_PtCl_6_), 3,3′,5,5′−tetramethylbenzidine (TMB, ≥99%), L−histidine (L−His), glutathione (GSH), glutamic acid (Glu), and fetal bovine serum were purchased from Macklin Biochemical Co., Ltd. (Shanghai, China). Sodium borohydride (NaBH_4_), disodium hydrogen phosphate (Na_2_HPO_4_), p−Benzoquinone (p−BQ), isopropanol (IPA), potassium nitrate (KNO_3_), citric acid, ascorbic acid (AA), and sodium nitrate (NaNO_3_) were purchased from Sinopharm Chemical Reagents Co., Ltd. (Shanghai, China).

### 2.2. Instruments

X-ray diffraction patterns (XRD) were obtained using a Rigaku Smartlab 3 KW X-ray diffractometer with Cu Kα radiation in the 2θ range from 10° to 80° at a scan rate of 10°/min. Scanning electron microscopy (SEM) images were captured using a ZEISS Sigma 300. X-ray photoelectron spectroscopy (XPS) analysis was performed on the Thermo Scientific K−Alpha (USA). A Bruker EMXplus−6/1 was used to record the electron paramagnetic resonance (EPR) spectra. Transmission electron microscopy (TEM) images were acquired from JEOL JEM 2100 (operational voltage of 200 kV). Absorbance was determined by a UV−8000 ultraviolet−visible (UV−vis) spectrophotometer. The pH value of the buffer solution was measured using a PHS−3D pH meter.

### 2.3. Synthesis of TiO_2_

Firstly, TiO_2_ microspheres were synthesized by following the literature−reported procedure [16]. Specifically, 10 mL tetrabutyltitanate was added to 3 mL concentrated hydrochloric acid and stirred for 15 min. 2 mL TiCl_4_ (2 M) aqueous solution was dropped into the mixture within 10 min. Then, 50 mL of methylbenzene was added to the uniform mixture and stirred for 2 h. The mixture was then placed in a Teflon−lined autoclave at 150 °C for 4 h. Finally, it was washed several times with deionized water and ethanol and dried at 60 °C for 6 h.

### 2.4. Synthesis of BaTiO_3_ SUMs

Firstly, 0.32 g TiO_2_ were dispersed in 40 mL ultra−pure water and mixed with 40 mL 2.52 g Ba(OH)_2_·8H_2_O aqueous solution. The mixture was transferred to 100 mL Teflon−lined autoclave after stirring for 30 min and heated at 160 °C for 6 h. To remove any particles (if formed) of barium carbonate byproducts after hydrothermal conversion while the suspension is still hot (~60 °C), 10 mL of formic acid is added and precipitated at room temperature for 15 min. The precipitate was washed several times with deionized water and ethanol and dried at 60 °C for 6 h.

### 2.5. Synthesis of AuPt@BaTiO_3_ SUMs

Initially, 0.2 g BaTiO_3_ was dispersed in 60 mL of ultra−pure water. Then, H_2_PtCl_6_ (2 mM) and HAuCl_4_·3H_2_O (2 mM) were added to the solution and stirred for 2 h. During the stirring, 2 mL NaBH_4_ solution (0.1 mol L^−1^) was added and the agitation continued for 4 h. The suspension was observed to change from pale yellow to gray−black. The precipitate was collected by centrifugal separation and washed three times with deionized water and ethanol. Finally, it was dried at 60 °C for 6 h. Au@BaTiO_3_ SUMs and Pt@BaTiO_3_ SUMs were prepared by adding HAuCl_4_·3H_2_O and H_2_PtCl_6_, respectively. The remaining steps were the same as the above process.

### 2.6. Catalytic Mechanism of Oxidase−like Activity

To study the reactive oxygen species (ROS) of AuPt@BaTiO_3_ SUMs oxidase−like activity, a series of comparative experiments were carried out with different ROS scavengers and the removal of dissolved oxygen in water. ROS scavengers L−histidine, p−Benzoquinone (p−BQ), isopropanol (IPA), and EDTA−2Na were added to capture singlet oxygen (^1^O_2_), superoxide radicals (O_2_^•−^), hydroxyl radicals (·OH) and hole (h^+^), respectively. Specifically, the AuPt@BaTiO_3_ SUMs solution (0.28 mg mL^−1^) and scavenger (5 mM) were added to the Na_2_HPO_4_−CA buffer (0.5 mM TMB, pH = 4.0, 0.2 M). Then, the solution was reacted at 20 °C for 10 min and the absorbance was measured. Furthermore, the Na_2_HPO_4_−CA buffer (0.5 mM TMB, pH = 4.0, 0.2 M) was bubbled with N_2_ for 10 min to remove dissolved oxygen. Then, AuPt@BaTiO_3_ SUMs solution (0.28 mg mL^−1^) was added to the buffer solution, and the absorbance was measured after reaction at 20 °C for 10 min. The mechanism of the AuPt@BaTiO_3_ SUMs Photo−piezoelectric enhanced oxidase−like activity was investigated by comparative experiments. The AuPt@BaTiO_3_ SUMs solution (0.28 mg mL^−1^) was added to Na_2_HPO_4_−CA buffer (pH = 4.0, 0.2 M) containing 0.5 mM TMB. Then, the reaction was performed for 1 min under different conditions (dark, light, ultrasound, light−ultrasound), and the absorbance was measured.

### 2.7. Photo−Piezoelectric Enhanced AuPt@BaTiO_3_ SUMs Oxidase−like Activity

TMB was used as a colorimetric substrate to demonstrate the oxidase−like activity of AuPt@BaTiO_3_ SUMs. Usually, AuPt@BaTiO_3_ SUMs (0.28 mg mL^−1^) were added to the Na_2_HPO_4_−CA buffer solution (0.5 mM TMB, pH = 4.0, 0.2 M). The absorbance of the solution was measured after reaction for 1 min under different conditions.

### 2.8. Kinetic Analysis

The concentration of TMB changed from 0.1 mM to 1.0 mM, and the absorbance curve of 60 s reaction was recorded at 20 °C. The initial reaction rate (V) of different substrate concentrations was obtained by Equation (1) [17]:

(1) $$V=\frac{k}{\varepsilon b}\;(\varepsilon =3.9\times 10^{4}\;M^{-1}\cdot {\mathrm{cm}}^{-1},\;b=1\mathrm{cm},\;k=\Delta A\;{\Delta t}^{-1}).$$

V_max_ and K_m_ were calculated by the Michaelis−Menten equation and the Lineweaver−Burk plot (Equation (2)) [18]:

(2) $$\frac{1}{V}=\frac{K_{m}}{V_{max}}(\frac{1}{\left[S\right]})+\frac{1}{V_{max}}$$

where K_m_ was the Michaelis constant, V was the initial velocity, [S] was the concentration of the substrate, and Vmax was the maximum reaction velocity [19].

### 2.9. Colorimetric Detection of GSH

GSH solution of varying concentrations was added under optimal reaction conditions. The absorbance was measured at 20 °C for 10 min (after exposure to light−ultrasound for 3 min). The selectivity of the AuPt@BaTiO_3_ SUMs colorimetric platform was assessed using interfering materials as controls.

The selectivity of colorimetric detection was investigated, using interference substances as controls. Among them, the concentrations of L−His, Gly, Leu, Arg, Lys, Pro, Glu, K^+^, Na^+^, Cys, mixture, and AA were 80 μM.

Fetal bovine serum was selected as the real sample for the detection of GSH content. (1) The fetal bovine serum was diluted 1000 times. Then, GSH concentration was detected by the method described above.

## 3. Results

### 3.1. Characterization of the AuPt@BaTiO_3_ SUMs

**Figure 1:**
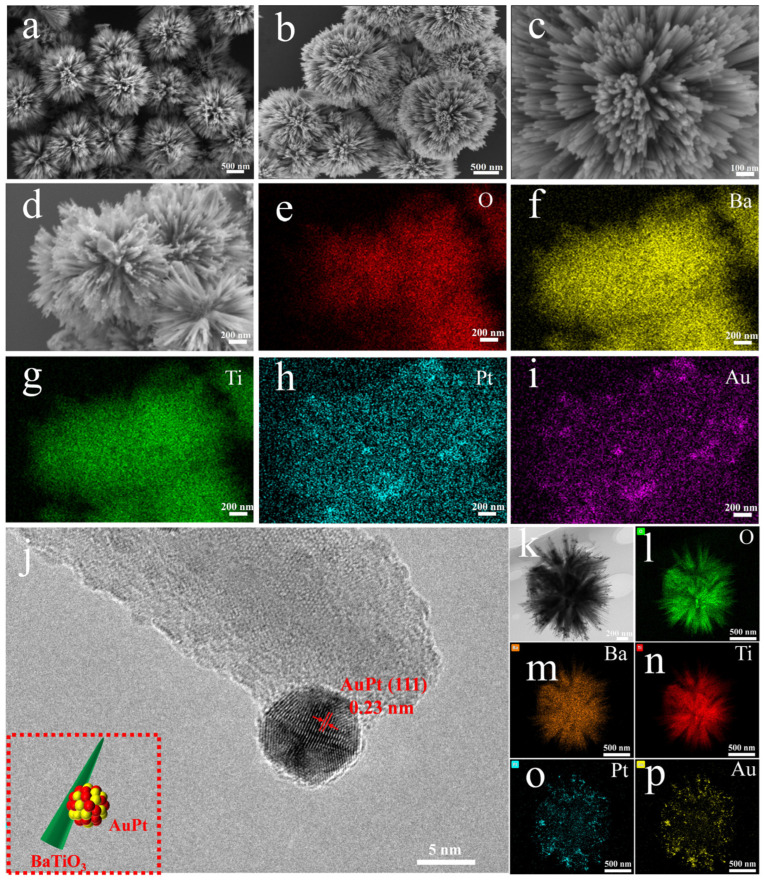
SEM image of (**a**) TiO_2_, (**b**–**c**) BaTiO_3_ SUMs, and (**d**) AuPt@BaTiO_3_ SUMs. (**e**–**i**) EDS mapping of the AuPt@BaTiO_3_ SUMs. (**j**–**k**) HRTEM and TEM images of the AuPt@BaTiO_3_ SUMs and (**l**–**p**) EDS mapping of the single AuPt@BaTiO_3_ SUMs. Inset: schematic illustration of the detailed structure of the AuPt@BaTiO_3_ SUMs.

BaTiO_3_ SUMs were fabricated via the hydrothermal synthesis of a TiO_2_ precursor. Their surface was subsequently modified with AuPt alloy nanoparticles using the chemical reduction method, as depicted in Figure S1. In Figure 1a,b, the synthesized 3D TiO_2_ and BaTiO_3_ SUMs exhibit a diameter of about 1 μm. The shape of the BaTiO_3_ SUMs, formed through the diffuse−reaction mechanism, remained almost unchanged from that of the precursor TiO_2_ [20]. Furthermore, the width of the BaTiO_3_ nanorods ranged between 5–40 nm, as seen in Figure 1c. In Figure 1d, we observed uniformly dispersed AuPt alloy nanoparticles on AuPt@BaTiO_3_ SUMs. TEM images, represented in Figure 1j,k, further confirmed the presence of these dispersed AuPt nanoparticles with diameters ranging from 5–25 nm. The HRTEM image in Figure 1j indicated a lattice spacing of 0.23 nm for the AuPt alloy nanoparticles, corresponding to the AuPt alloy (111) planes [21]. A bimetallic nanoparticle was prominently seen, beautifully adorned on the BaTiO3 nanorods. EDX analysis, shown in Figure S2, along with element mapping (Figure 1e–i,l–p, and Figure S3), further corroborated the uniform distribution of AuPt alloy nanoparticles on the BaTiO_3_ SUMs’ surface.

XRD diffraction patterns of the obtained product are presented in Figure S4. The TiO_2_ sample exhibited diffraction peaks at 2θ values of 26.5°, 33.7°, 36.0°, 37.6°, 51.5°, 54.4°, 61.5°, 62.8°, and 65.5°. These correspond to the (110), (101), (111), (210), (211), (220), (002), (310), and (301) crystal planes of the tetragonal rutile phase of TiO_2_ (JCPDS 21−1276) [22]. Moreover, sharp diffraction peaks observed in the 2θ range from 10° to 80° align perfectly with the structural crystals of the tetra system barium perovskite titanate (JCPDS 05−0626) [23]. Peaks observed at approximately 38.8°, 45.0°, and 65.7° can be attributed to the (111), (200), and (220) planes of the AuPt alloy, signaling the formation of the AuPt alloy [24].

**Figure 2:**
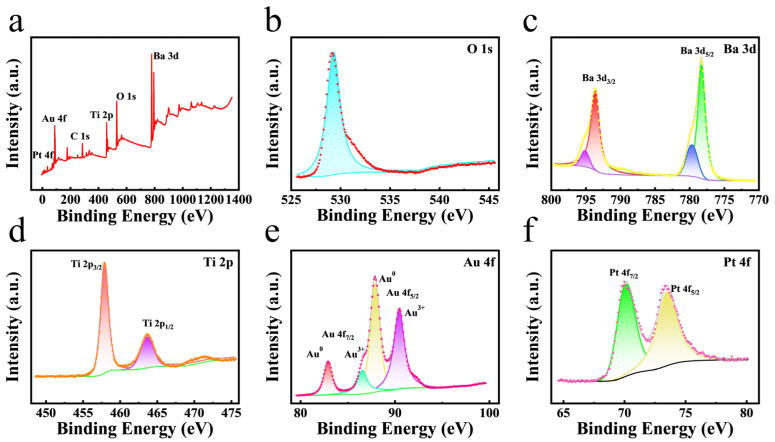
(**a**) Survey spectra of the AuPt@BaTiO_3_ SUMs. (**b**–**f**) High−resolution XPS spectra for O 1s, Ba 3d, Ti 2p, Au 4f, and Pt 4f.

The elemental chemical states in the AuPt@BaTiO_3_ SUMs were analyzed using XPS. Figure 2a reveals the presence of five elements: O, Ba, Ti, Au, and Pt in the sample. The high−resolution XPS spectrum for O 1s in the AuPt@BaTiO_3_ SUMs, displayed in Figure 2b, showcases the O 1s signal at 529.30 eV, devoid of any satellite peaks, indicative of the high crystallinity of the AuPt@BaTiO_3_ SUMs [25]. In Figure 2c, two peaks at 778.29 eV and 793.58 eV are associated with Ba 3d_5/2_ and Ba 3d_3/2_ in the perovskite structure, respectively. Another pair of peaks at 779.64 eV and 795.10 eV relate to Ba atoms in non−perovskite structures [26]. The deconvoluted Ti 2p spectrum, as shown in Figure 2d, highlights two predominant peaks: Ti 2p_3/2_ at 457.87 eV and Ti 2p_1/2_ at 463.59 eV, typically denoting Ti^4+^ [27]. The high−resolution map of Au 4f, displayed in Figure 2e, indicates that Au° (at 82.88 and 87.89 eV) and Au^3+^ (at 86.55 and 90.42 eV) correspond to Au 4f_7/2_ and Au 4f_5/2_, respectively [28]. The deconvoluted Pt 4f spectrum in Figure 2f shows main peaks at 70.10 eV and 73.48 eV, designated for Pt 4f_7/2_ and Pt 4f_5/2_, respectively [29]. The electron interaction within the alloy is evident by the negative shift in the Pt 4f peaks (from 75.8 eV to 72.5 eV) when compared to monometallic Pt, further confirming the formation of the AuPt alloy. These collective findings affirm the successful synthesis of AuPt@BaTiO_3_ SUMs.

### 3.2. Catalytic Mechanism of Oxidase−like Activity

The oxidase−like activity was examined under varying parameters: nano−enzyme concentration, pH value, temperature, and reaction time. Optimal conditions were observed when the pH was set to 4 (Figure S5), the reaction temperature was 20 °C (Figure S6), the reaction time was 10 min (Figure S7), and the AuPt@BaTiO_3_ SUMs concentration was 0.28 mg mL^−1^ (Figure S8). Further, under light−ultrasound conditions, the ideal illumination time for the oxidase mimetics of AuPt@BaTiO_3_ SUMs was determined to be 3 min (Figure S9).

**Figure 3:**
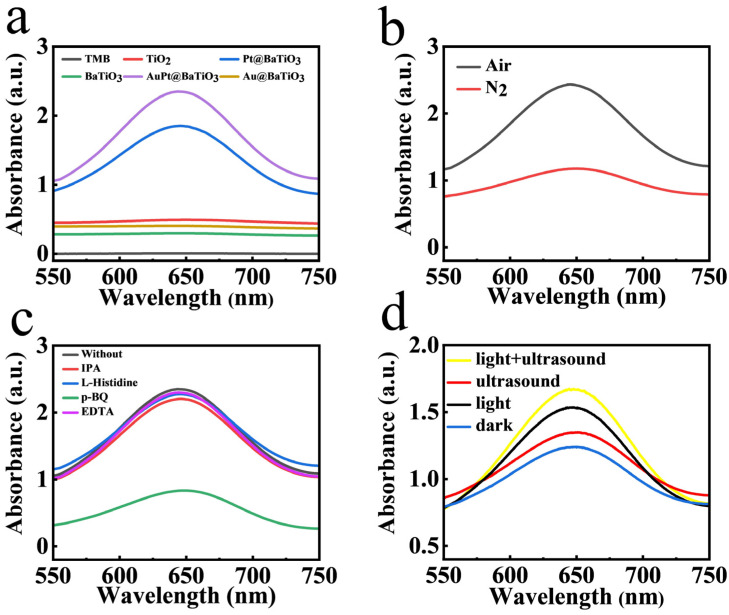
(**a**) UV−vis spectra of different reaction systems. (**b**) UV−vis spectra under different gaseous conditions (Air, N_2_). (**c**) UV−vis spectra of different capture agents for the (AuPt@BaTiO_3_ SUMs + TMB) system. (**d**) UV−vis spectra of the (AuPt@BaTiO_3_ SUMs + TMB) system under different conditions (dark, light, ultrasound, light−ultrasound).

Figure 3a illustrates that the absorption peak of AuPt@BaTiO_3_ SUMs was markedly higher than that of Au@BaTiO_3_ SUMs and Pt@BaTiO_3_ SUMs. This establishes that the AuPt@BaTiO_3_ bimetallic alloy Schottky junction substantially augmented the oxidase−like activity.

The oxidase−like activity mechanism of AuPt@BaTiO_3_ SUMs was explored in different atmospheres. According to Figure 3b, the absorbance post N_2_ bubbling was significantly reduced compared to that in air. This underscores the dependence of oxidase−like activity on dissolved oxygen. It is postulated that the dissolved oxygen may transform into reactive oxygen species (ROS) during oxidation, producing O_2_^•−^, ·OH, ^1^O_2_, and h^+^. To further this understanding, scavengers like 5 mM p−BQ, IPA, L−histidine, and EDTA were introduced to counteract O_2_^•−^, ·OH, ^1^O_2_, and h^+^, respectively [30]. Figure 3c shows that, except for HQ which drastically decreased absorbance, the other scavengers had minimal impact. This infers a pivotal role for O_2_^•−^ in the catalytic oxidation process.

To delve deeper into the catalytic mechanism, electron paramagnetic resonance (EPR) tests were executed using 5, 5−dimethyl−1−pyrroline N−oxide (DMPO). Figure S10 reveals a characteristic peak associated with O_2_^•−^. Notably, with prolonged illumination time, this signal’s intensity increased, corroborating the notion that light considerably elevates the O_2_^•−^ quantity.

Figure 3d highlights that the absorption peak intensity was amplified under either ultrasound or light conditions compared to darkness. Intriguingly, a combination of light–ultrasound further intensified this peak. While it is recognized that BaTiO_3_ SUMs generate a polarization charge under ultrasonic cavitation’s mechanical stress with suboptimal catalytic efficiency [31], the combined light–ultrasound stimulation significantly boosts this efficiency. This suggests a synergistic enhancement in oxidase−like activity due to combined light–ultrasound action.

**Scheme 1:**
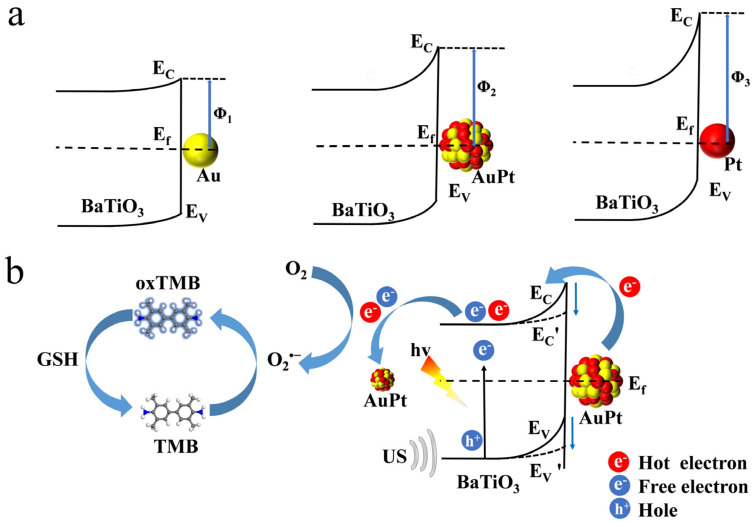
(**a**) Schematic illustration of energy−band structures for Au@BaTiO_3_ SUMs, Pt@BaTiO_3_ SUMs, and AuPt@BaTiO_3_ SUMs. (**b**) The photo−piezoelectric effect enhances the oxidase−like activity of the AuPt@BaTiO_3_ SUMs.

As illustrated in Scheme 1, the augmented oxidase−like activity stems from the photo−piezoelectric effect of the AuPt@BaTiO_3_ bimetallic alloy Schottky junction. The main factors contributing to this increase are:

1. Formation of the Bimetallic Alloy Schottky Junction: AuPt alloy nanoparticles were integrated onto BaTiO_3_ SUMs, establishing a bimetallic alloy Schottky junction. With BaTiO_3_ SUMs’s work function at 4.8 eV and the AuPt alloy’s work function lying between that of Au (5.1 eV) and Pt (5.65 eV) [32,33,34], AuPt@BaTiO_3_ SUMs create an optimal Schottky barrier between Au@BaTiO_3_ SUMs and Pt@BaTiO_3_ SUMs. As illustrated in Scheme 1a, Pt@BaTiO_3_ SUMs only allow a limited number of photoexcited electrons to traverse the elevated Schottky barrier Φ_3_. The relatively low barrier height Φ_1_ of Au@BaTiO_3_ SUMs also allowed the backflow of photogenerated electrons, resulting in the recombination of charge carriers [35]. Conversely, the lower barrier height Φ_2_ in AuPt@BaTiO_3_ SUMs promotes more substantial charge separation, facilitating the transfer of photogenerated electrons [36].

2. Positive Polarization Charge Under Ultrasound: The polarization charge that arises at the interface under ultrasound induces a downward bend in the BaTiO_3_ SUMs’ energy band [31]. This reduced energy band height expedites the migration of photogenerated electrons to AuPt alloy nanoparticles [14,37]. Concurrently, the energy threshold for hot electrons to cross this barrier is diminished, bolstering the efficient separation of hot electrons that arise due to the excitation of larger AuPt alloy nanoparticles [38,39]. Moreover, the more compact AuPt alloy nanoparticles act as electron sinks, capturing excited electrons, which accelerates surface electron migration, thereby amplifying enzyme activity [40].

3. Synergistic Effect of Au and Pt Alloying: The amalgamation of Au and Pt offers a potent synergistic effect, primarily driven by the alteration of the d−band center and kinetics. The alloying triggers a downward shift of Pt’s d−band center, which attenuates the binding between Pt and oxygen, thereby enhancing catalytic activity [41,42].

In summary, factors like the Schottky junction, LSPR effect, and piezoelectric effect collectively enhance photosensitive carrier separation. This optimized separation facilitates the increased production of O_2_^•−^, culminating in heightened oxidase activity.

### 3.3. Steady−State Kinetics

Steady−state kinetic experiments were carried out using varying concentrations of TMB, ranging from 0.1 to 1.0 mM, to examine the oxidase−like activity of AuPt@BaTiO_3_ SUMs (Table S1). The Michaelis constant (K_m_) and the maximum reaction velocity (V_max_) were derived from the Michaelis–Menten curve (as depicted in Figure S11b) and the double reciprocal (Lineweaver–Burk) plot (presented in Figure S11c). The obtained values were 0.1218 mM for K_m_ and 3.16 × 10^−7^ M s^−1^ for V_max_, respectively. It is well−established in enzymology that a lower K_m_ value signifies a greater substrate affinity, while a higher V_max_ points towards a rapid enzymatic response. A comparison with data from previously reported studies, as presented in Table 1, reveals that the oxidase mimetics of AuPt@BaTiO_3_ SUMs demonstrate superior affinity towards the substrate TMB. Moreover, the reaction rate was found to be more rapid. Consequently, this underscores the impressive catalytic efficiency of the AuPt@BaTiO_3_ SUMs.

### 3.4. GSH Detection

**Figure 4:**
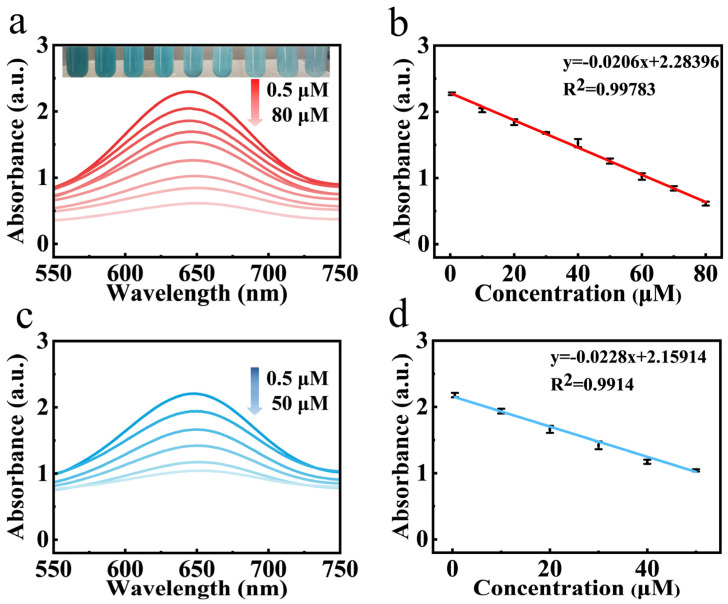
(**a**) UV−vis absorbance of the detection system upon the addition of 0.5–80 μM GSH in the dark. (**b**) The calibration curve corresponds to GSH concentration. Inset: the corresponding photograph of the colored products for reaction with different GSH concentrations. Reaction conditions: 0.5 mM TMB, 0.28 mg mL^−1^ AuPt@BaTiO_3_ SUMs, 0.2 M Na_2_HPO_4_−CA buffer (pH = 4.0), 10 min and 20 °C. (**c**) UV−vis absorbance of the detection system upon the addition of 0.5–50 μM GSH under light–ultrasound. (**d**) The calibration curve corresponds to GSH concentration. Reaction conditions: 0.5 mM TMB, 0.28 mg mL^−1^ AuPt@BaTiO_3_ SUMs, 0.2 M Na_2_HPO_4_−CA buffer (pH = 4.0), 3 min, and 20 °C.

As an antioxidant, GSH effectively consumes O_2_^•−^ and reduces oxTMB to TMB via a single electron transfer process [50]. The absorbance at 652 nm was observed to decrease progressively with increasing GSH concentration (Figure 4a,c). The standard curve was plotted based on the relationship between the peak absorption intensity and GSH concentration. In the dark, the linear equation is: Abs = −0.0206C_[GSH]_ + 2.284 (R^2^ = 0.9978) (Figure 4b). Under light−ultrasound conditions, the linear equation is Abs = −0.0228C_[GSH]_ + 2.159 (R^2^ = 0.9914) (Figure 4d). The reaction times were 10 min in the dark and 3 min under light–ultrasound. The LOD was determined to be 0.415 μM in the dark and 0.225 μM under light−ultrasound, both of which are lower than the values reported in previous studies (Table 2).

**Figure 5:**
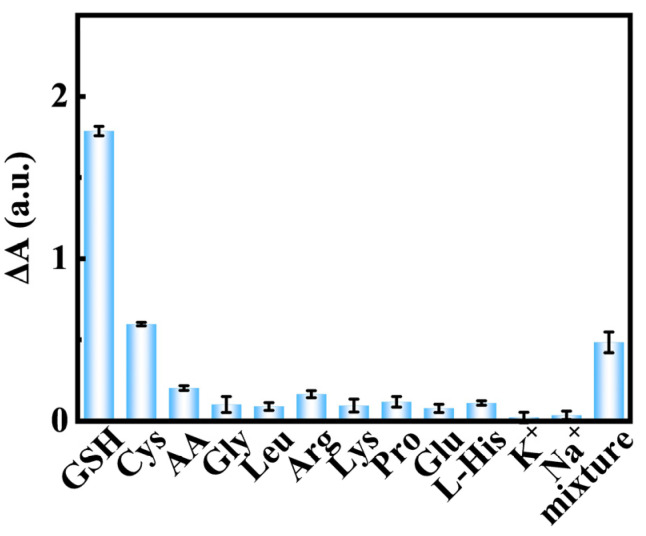
Anti−interference experiments of the colorimetric detection of GSH (80 μM GSH, mixture, Cys, AA, Gly, Leu, Arg, Lys, Pro, Glu, L−His, K^+^, Na^+^).

Possible disruptors in human serum were examined to assess the system’s selectivity. The results (Figure 5) indicated that Cys did interfere with the system’s selectivity to a degree, but the interference from other disruptors was minimal. Given that the Cys concentration in human serum is significantly lower than the GSH concentration [58,59], this interference is limited. Thus, the colorimetric detection of GSH demonstrated excellent selectivity.

Furthermore, stability is a crucial factor when evaluating potential applications. The oxidase−like activity of AuPt@BaTiO_3_ SUMs was tested every 5 min, revealing impressive short−term stability, with a relative standard deviation (RSD) of 1.46% after 11 cycles (Figure S12a). Simultaneously, the absorbance measured every alternate day displayed minor fluctuations in catalytic performance over 15 days, boasting an RSD of 0.63%, indicative of commendable long−term stability (Figure S12b). To evaluate repeatability, five batches of AuPt@BaTiO_3_ SUMs oxidase mimetics were prepared under identical experimental conditions. The nearly consistent absorbance results across batches suggest excellent reproducibility (Figure S13).

To assess the accuracy of the colorimetric determination of GSH in serum, the GSH content in real samples was evaluated using the standard addition method [60]. The recovery rate for fetal bovine serum ranged from 99.91% to 101.8%, with an RSD of less than 1.06%. These results suggest that this method holds promise for detecting GSH in real−world settings (Table 3).

## 4. Conclusions

In conclusion, we successfully prepared AuPt@BaTiO_3_ SUMs, which showcased outstanding oxidase−like activity to serve as oxidase mimetics for the colorimetric detection of GSH. The combined effects of the photo−piezoelectric phenomenon and LSPR facilitated the directed migration and separation of photogenerated electrons, enhanced by the AuPt bimetallic alloy. This photo−piezoelectric influence significantly boosted oxidase−like activity, leading to high−performance colorimetric GSH detection. Remarkably, our detection platform’s superior catalytic activity allowed for a short detection time of just 3 min, ensuring high sensitivity with a linear range of 0.5–50 μM and an impressive LOD of 0.225 μM for GSH detection. Furthermore, this sensing approach demonstrated commendable analytical performance in intricate samples, boasting a reliable recovery rate of 99.91–101.8% in real−world sample testing. Overall, this research introduces a novel perspective on the strategic design of highly efficient nanozymes leveraging the photo−piezoelectric effect.

## Figures and Tables

**Table 1:** Comparison of catalytic kinetic parameters.

Materials	Substrate	V_max_ (×10^−7^ M s^−1^)	K_m_ (mM)	Reference
HRP	TMB	1	0.43	[43]
CS@Pt NS	TMB	6.524	0.49	[44]
3:3−Mn_3_O_4_@g−C_3_N_4_	TMB	1.645	0.343	[45]
PtPdNPs@g−C_3_N_4_	TMB	3.639	0.225	[46]
BiO−BDC−NH_2_	TMB	0.581	0.41	[47]
Fe−MOF	TMB	0.56	2.6	[48]
DSMIP@Mn_3_O_4_	TMB	0.092	5.1	[49]
AuPt@BaTiO_3_ SUMs	TMB	3.16	0.1218	This work

**Table 2:** Comparison of the sensing performance with other methods in the detection of GSH.

Materials	Methods	Linear Range (μM)	LOD (μM)	Detection Time (min)	Ref.
ox−MoS_2_ NSs	colorimetry	0.5–5	0.276	60	[51]
Por−ZnFe_2_O_4_/rGO	colorimetry	2–40	0.76	10	[52]
hemin−PNT	colorimetry	1–35	0.51	10	[53]
TTz−Cl^2+^	colorimetry	1–17	0.47	15	[54]
AgNPs/C–MWCNT/PANI/Au	electrochemistry	0.3–0.35	0.3	−	[55]
CDs−MnO_2_ NFs	fluorescence	2–200	0.558	5	[56]
Cys−MnO_2_ nanospheres/MnO_2_ nanosheets	fluorescence	5–50,150–800	2.96	15	[57]
AuPt@BaTiO_3_ SUMs	colorimetry	0.5–50	0.225	3	This work

**Table 3:** Detection of GSH in an actual sample (*n* = 3).

Sample	Conditions	Added (μM)	Found (μM)	Recovery (%)	RSD (%)
fetal bovine serum	light−ultrasound	10	10.18	101.8	0.37
		30	29.97	99.91	0.39
		50	50.66	101.3	1.06

## Data Availability

Data are contained within the article.

## Supplementary Materials

The following supporting information can be downloaded at: https://www.mdpi.com/article/10.3390/s24072242/s1, Figure S1: Fabrication process of the AuPt@BaTiO_3_ SUMs; Figure S2: EDX photo of the prepared AuPt@BaTiO_3_ SUMs; Figure S3: EDS mapping of the AuPt@BaTiO_3_ SUMs; Figure S4: XRD patterns of the TiO_2_, BaTiO_3_ SUMs and the AuPt@BaTiO_3_ SUMs; Figure S5: The oxidase−like activity of AuPt@BaTiO_3_ SUMs depends on pH. Reaction condition: 0.28 mg mL^−1^ AuPt@BaTiO_3_ SUMs, 0.2 M Na_2_HPO_4_−CA buffer, 0.5 mM TMB, 10 min, 20 °C; Figure S6: The oxidase−like activity of AuPt@BaTiO_3_ SUMs depends on temperature. Reaction condition: 0.28 mg mL^−1^ AuPt@BaTiO_3_ SUMs, 0.2 M Na_2_HPO_4_−CA buffer (pH=4.0), 0.5 mM TMB, 10 min; Figure S7: Time−dependent absorbance changes of TMB oxidation at 652 nm. Reaction condition: 0.28 mg mL^−1^ AuPt@BaTiO_3_ SUMs, 0.2 M Na_2_HPO_4_−CA buffer (pH=4.0), 0.5 mM TMB, 20 °C; Figure S8: The concentration effects on the oxidase−like activity of the AuPt@BaTiO_3_ SUMs. Reaction condition: 0.2 M Na_2_HPO_4_−CA buffer (pH=4.0), 0.5 mM TMB, 10 min, 20 °C; Figure S9: Time−dependent absorbance changes of TMB oxidation at 652 nm. Reaction condition: 0.28 mg mL^−1^ AuPt@BaTiO_3_ SUMs, 0.2 M Na_2_HPO_4_−CA buffer (pH=4.0), Hg lamp and ultrasound, 0.5 mM TMB, 20 °C; Figure S10: DMPO−EPR spin−trapping spectra for O_2_^•−^; Figure S11: Steady−state kinetics of TMB oxidation using the AuPt@BaTiO_3_ SUMs: (a) Typical absorbance spectra of different reaction systems for 1 min. (b) Michaelis−Menten curves for different TMB concentrations. (c) Lineweaver−Burk plot for different TMB concentrations; Figure S12: (a) The short−term stability of the AuPt@BaTiO_3_ SUMs. (b) The long−term storage stability of the AuPt@BaTiO_3_ SUMs; Figure S13: The reproducibility of the AuPt@BaTiO_3_ SUMs; Table S1: Data of catalytic kinetic parameters.
