# Supplementary material for: Using the Photo–Piezoelectric Effect of AuPt@BaTiO3 Oxidase Mimetics for Colorimetric Detection of GSH in Serum
Source: Sensors (Basel). 2024 Mar 31;24(7):2242. doi: 10.3390/s24072242 (PMC11014263; doi:10.3390/s24072242)
Supplement: Supplementary file 1 [file sensors-24-02242-s001.zip › sensors-2865734-supplementary.pdf]

sensors

Supporting Information

Using the Photo-Piezoelectric Effect of AuPt@BaTiO_3 Oxidase Mimetics for Colorimetric Detection of GSH in Serum

Yiquan Liao, Yichang He, Bin Zhang, Ye Ma *, Ruiqi Xu, Minggang Zhao * and Hongzhi Cui

School of Materials Science and Engineering, Ocean University of China, 266100 Qingdao, China

* Correspondence: maye@ouc.edu.cn (Y.M.); zhaomg@ouc.edu.cn (M.Z.)

Table of contents

1. Fabrication process of nanozymes 3

2. EDX 3

3. EDS mapping 3

4. XRD 4

5. Optimum condition 4

6. DMPO-EPR 7

7. Steady-state kinetics 7

8. Stability 8

1. Fabrication process of nanozymes

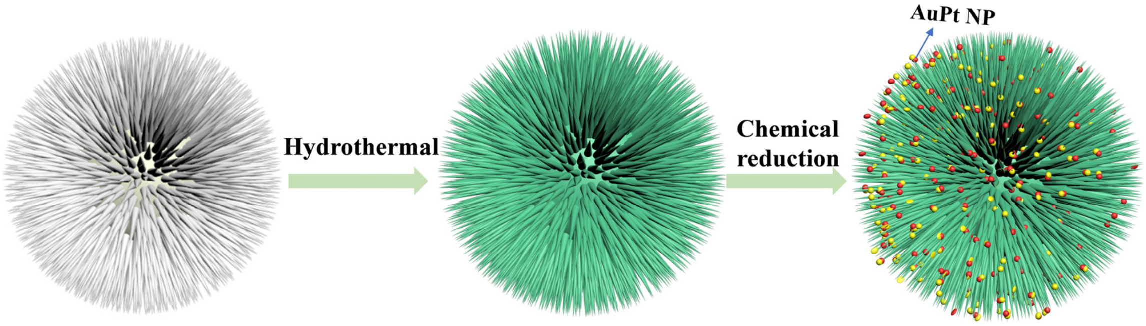

Figure S1. Fabrication process of the AuPt@$BaTiO_3$ SUMs.

2. EDX

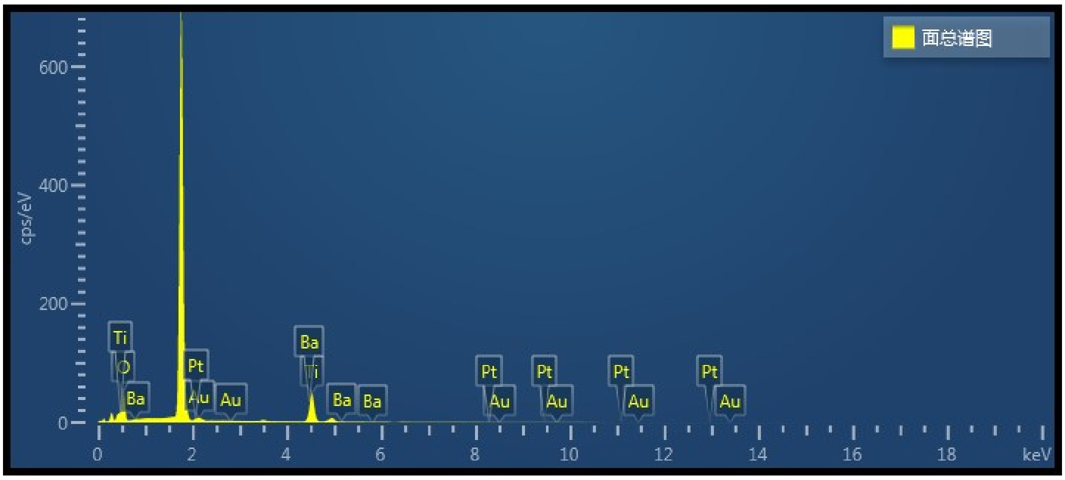

Figure S2. EDX photo of the prepared AuPt@$BaTiO_3$ SUMs.

3. EDS mapping

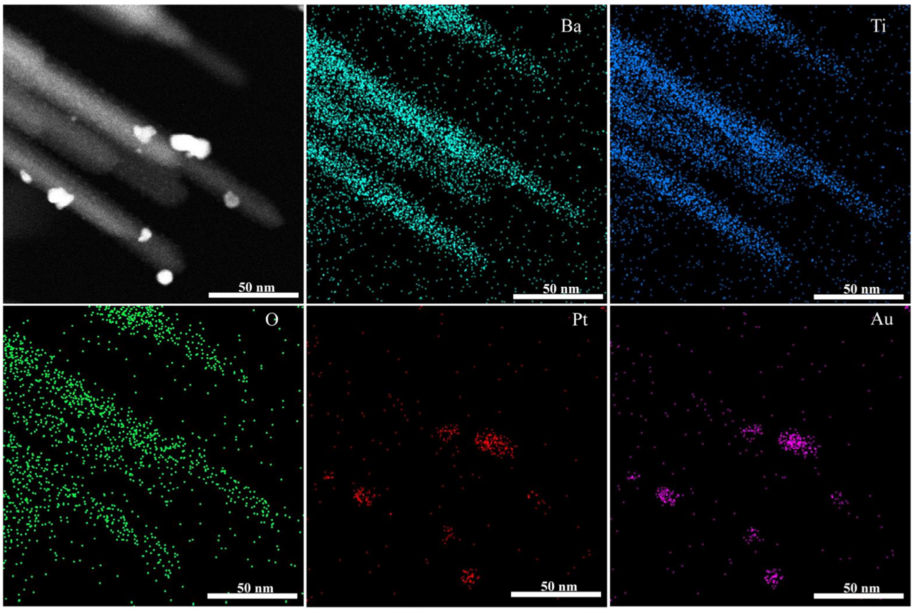

Figure S3. EDS mapping of the AuPt@$BaTiO_3$ SUMs.

4. XRD

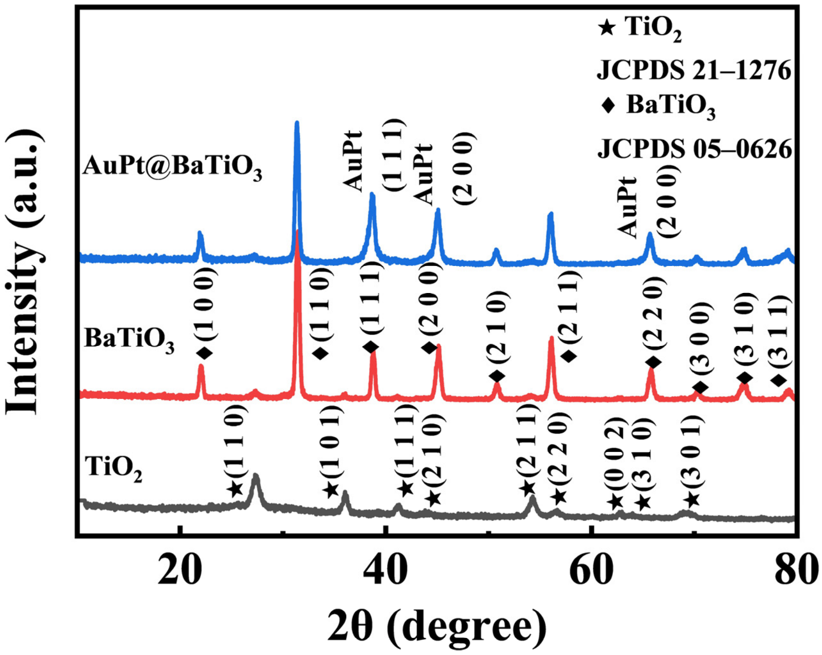

Figure S4. XRD patterns of the TiO_2, $BaTiO_3$ SUMs, and the AuPt@$BaTiO_3$ SUMs.

5. Optimum condition

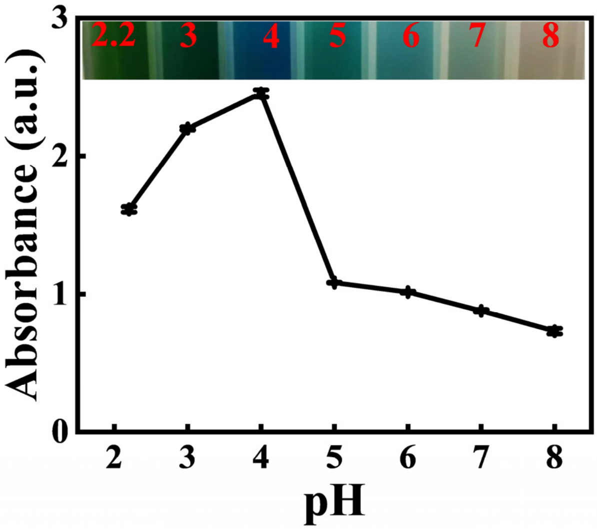

Figure S5. The oxidase-like activity of AuPt@$BaTiO_3$ SUMs depends on pH. Reaction condition: 0.28 mg mL^{-1} AuPt@$BaTiO_3$ SUMs, 0.2 M Na_2HPO_4-CA buffer, 0.5 mM TMB, 10 min, and 20 °C.

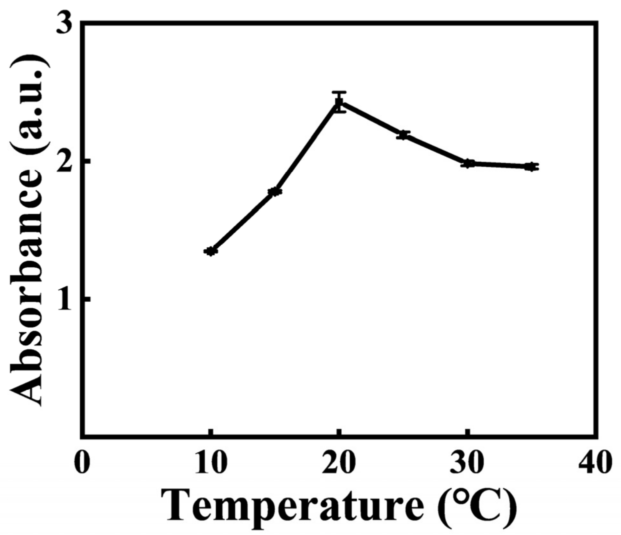

Figure S6. The oxidase-like activity of AuPt@$BaTiO_3$ SUMs depends on temperature. Reaction condition: 0.28 mg mL^{-1} AuPt@$BaTiO_3$ SUMs, 0.2 M Na_2HPO_4-CA buffer (pH=4.0), 0.5 mM TMB, and 10 min.

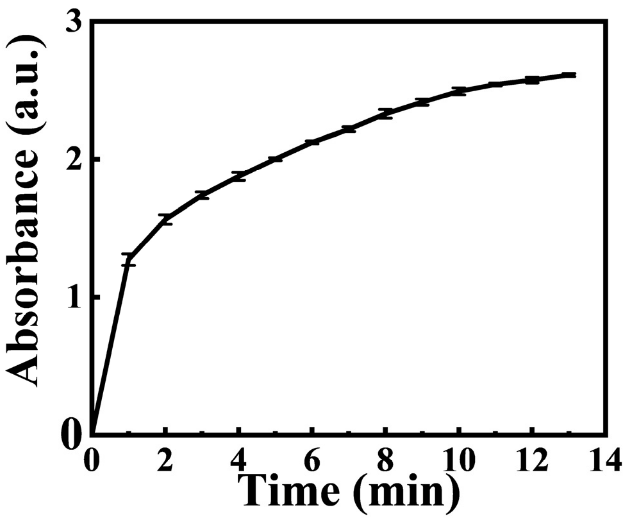

Figure S7. Time-dependent absorbance changes of TMB oxidation at 652 nm. Reaction condition: 0.28 mg mL^{-1} AuPt@$BaTiO_3$ SUMs, 0.2 M Na_2HPO_4-CA buffer (pH=4.0), 0.5 mM TMB, and 20 °C.

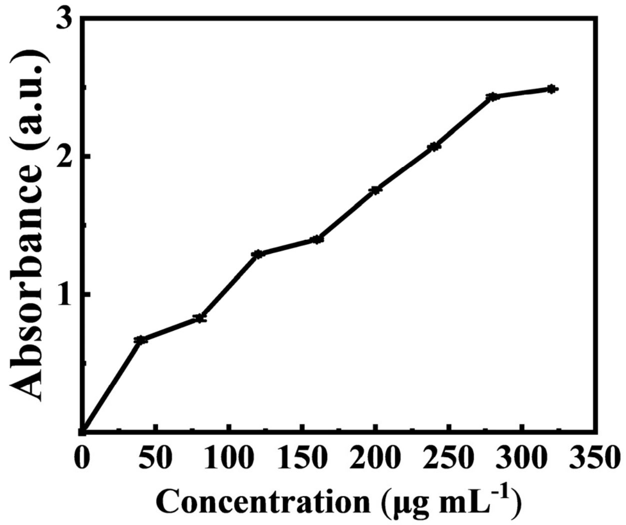

Figure S8. The concentration effects on the oxidase-like activity of the AuPt@$BaTiO_3$ SUMs. Reaction condition: 0.2 M Na_2HPO_4-CA buffer (pH=4.0), 0.5 mM TMB, 10 min, and 20 °C.

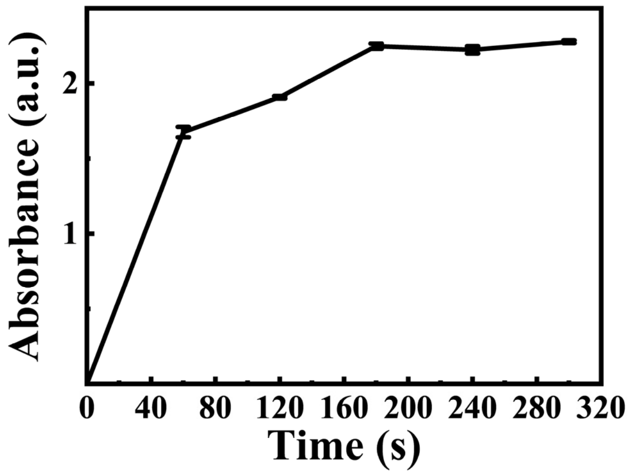

Figure S9. Time-dependent absorbance changes of TMB oxidation at 652 nm. Reaction condition: 0.28 mg mL^{-1} AuPt@$BaTiO_3$ SUMs, 0.2 M Na_2HPO_4-CA buffer (pH=4.0), Hg lamp and ultrasound, 0.5 mM TMB, and 20 °C.

6. DMPO-EPR

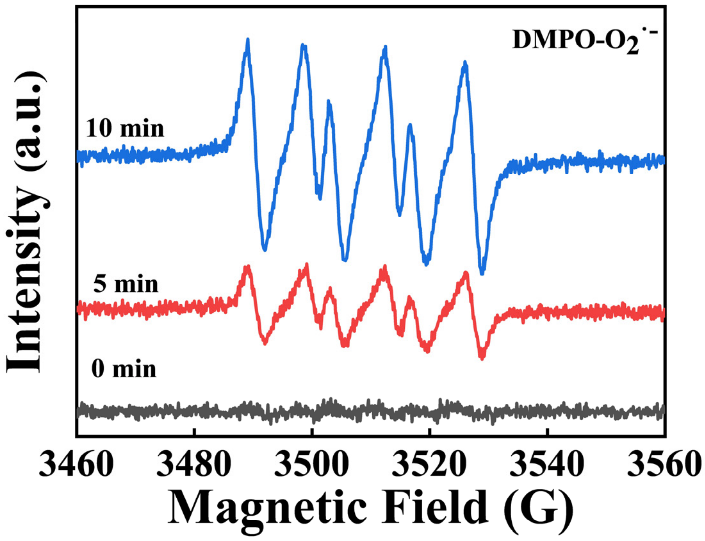

Figure S10. DMPO-EPR spin-trapping spectra for $O_2^{\bullet-}$.

7. Steady-state kinetics

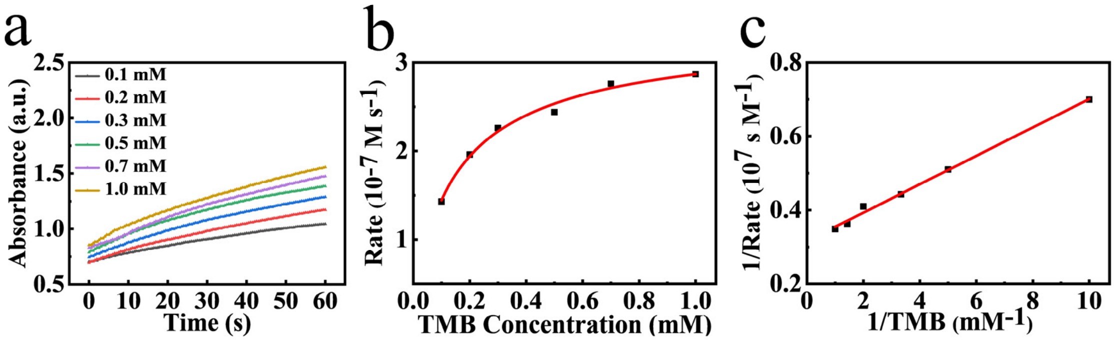

Figure S11. Steady-state kinetics of TMB oxidation using the AuPt@$BaTiO_3$ SUMs: (a) Typical absorbance spectra of different reaction systems for 1 min. (b) Michaelis–Menten curves for different TMB concentrations. (c) Lineweaver–Burk plot for different TMB concentrations.

8. Stability

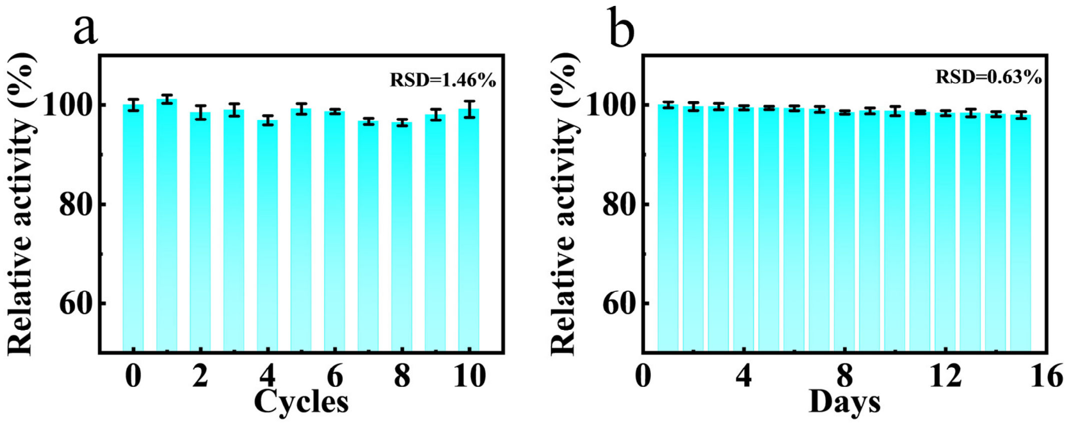

Figure S12. (a) The short-term stability of the AuPt@$BaTiO_3$ SUMs. (b) The long-term storage stability of the AuPt@$BaTiO_3$ SUMs.

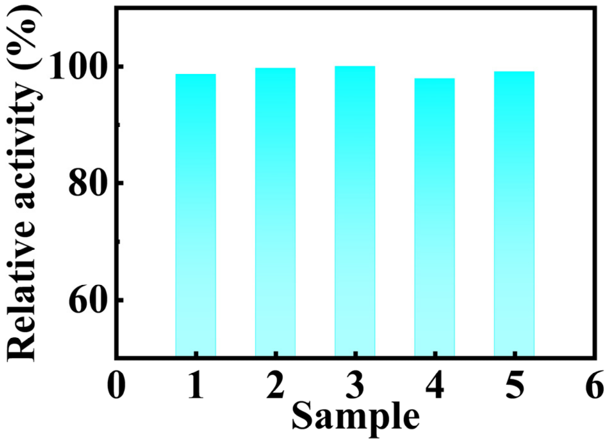

Figure S13. The reproducibility of the AuPt@$BaTiO_3$ SUMs.

Table S1. Data of catalytic kinetic parameters.

TMB concentration (mM)	k	initial reaction rate (V)	Michaelis-Menten curve		Lineweaver-Burk	
			x	y	x	y
0.1	0.00558	1.43E-07	0.1	1.43	10	0.6993
0.2	0.00765	1.96E-07	0.2	1.96	5	0.5102
0.3	0.00883	2.26E-07	0.3	2.26	3.333	0.4425
0.5	0.00953	2.44E-07	0.5	2.44	2	0.4098

0.7	0.01078	2.76E-07	0.7	2.76	1.428	0.3623
1.0	0.01119	2.87E-07	1.0	2.87	1	0.3484
